# A deep learning approach for fast muscle water T2 mapping with subject specific fat T2 calibration from multi-spin-echo acquisitions

**DOI:** 10.1038/s41598-024-58812-2

**Published:** 2024-04-08

**Authors:** Marco Barbieri, Melissa T. Hooijmans, Kevin Moulin, Tyler E. Cork, Daniel B. Ennis, Garry E. Gold, Feliks Kogan, Valentina Mazzoli

**Affiliations:** 1 Department of Radiology, Stanford University, Stanford, CA USA; 2 Department of Radiology and Nuclear Medicine, Amsterdam University Medical Center, Amsterdam, The Netherlands; 3 Department of Cardiology, Boston Children’s Hospital, Harvard Medical School, Boston, MA USA; 4 Department of Bioengineering, Stanford University, Stanford, CA USA; 5 Bernard and Irene Schwartz Center for Biomedical Imaging, Department of Radiology, New York University School of Medicine, New York, NY USA

**Keywords:** Magnetic resonance imaging, Biomedical engineering

## Abstract

This work presents a deep learning approach for rapid and accurate muscle water T_2_ with subject-specific fat T_2_ calibration using multi-spin-echo acquisitions. This method addresses the computational limitations of conventional bi-component Extended Phase Graph fitting methods (nonlinear-least-squares and dictionary-based) by leveraging fully connected neural networks for fast processing with minimal computational resources. We validated the approach through in vivo experiments using two different MRI vendors. The results showed strong agreement of our deep learning approach with reference methods, summarized by Lin’s concordance correlation coefficients ranging from 0.89 to 0.97. Further, the deep learning method achieved a significant computational time improvement, processing data 116 and 33 times faster than the nonlinear least squares and dictionary methods, respectively. In conclusion, the proposed approach demonstrated significant time and resource efficiency improvements over conventional methods while maintaining similar accuracy. This methodology makes the processing of water T_2_ data faster and easier for the user and will facilitate the utilization of the use of a quantitative water T_2_ map of muscle in clinical and research studies.

## Introduction

In understanding muscle health, the concept of "muscle quality" has become pivotal^1^. “Muscle quality” is a term encompassing the compositional and functional alterations occurring in skeletal muscles due to factors like aging, diseases, injury, disuse, or training. These changes, which go beyond mere "muscle quantity," are critical in assessing overall muscle health. In this context, Quantitative Magnetic Resonance Imaging (qMRI) has emerged as a leading technique for in vivo study of muscle health. Its capability to assess various biomarkers relevant to both "muscle quality" and "muscle quantity" across different skeletal sites makes it exceptionally valuable^2–4^.

A notable compositional alteration is the replacement of muscle fibers by fat, which is observed in various physiological and pathological conditions, such as aging^5,6^, muscle inactivity^7,8^, and neuromuscular disorders^3^. Fat infiltration, however, marks an irreversible stage in the degeneration of muscle tissue, limiting its utility to only indicating the chronic or end-stage of muscle degeneration. Thus, once fat infiltration is observed therapeutic interventions are limited and aimed at decelerating or halting the further deterioration of muscle tissue. However, preceding these irreversible changes, muscle fibers typically experience damage characterized by cycles of degeneration and regeneration. This ongoing damage often leads to persistent inflammation, contributing to the progression of muscular degeneration^9,10^. In this context, the transverse relaxation time (T_2_) of the water present in the myocytic tissue has been shown to be a valuable biomarker of disease activity. T_2_ has been shown to be sensitive to the inflammatory response indicative of early-stage muscular degenerative disorders, such as muscular dystrophies^11–13^, as well as conditions related to aging^14^ and injury^15^. Unlike fat fraction measurements, which reflect irreversible changes, T_2_ of the myocytic component provides information about processes that potentially can be reversed, offering valuable insights for timely and effective therapeutic interventions.

Two-dimensional Multi-Echo Spin-Echo (MESE) MRI is the gold standard method to obtain a quantitative measure of the T_2_ parameter. Typically, the experimental signal decay obtained from such sequences is analyzed voxel-wise using monoexponential modelling, which implicitly assumes that only a tissue component is present within an imaging voxel (“global T_2_”). This way, when multiple tissues are present within the voxels, they all contribute to the signal and therefore affect the measured T_2_. Inflammatory processes and fat replacement often co-exist within the same muscle, early detection of disease progression or response to treatments requires to separate the contribution of fat and muscle to the measured T_2_. When fat replacement (myosteatosis) is present, it results in a overestimation of T2 using a one component model for fitting. Elevations of T_2_, indicating disease activity^4^, are usually associated with myosteatosis, T_2_ becomes then a biomarker of fat infiltration hindering its ability to inform on disease activity. For this reason, the correct estimation of the T2 of lean muscle tissue (water T_2_ or T_2w_) requires multicomponent modeling. Furthermore, in 2D sequences the magnetic field used for excitation (B_1_^+^ field) generates an imperfect deposition of power across the slice thickness, the so-called slice profile effect. Thus, in MESE sequences this generates partial refocusing of the transverse magnetization across the slice. Contrary to perfect 180° refocusing pulses, pulses that are not exactly 180° not only partially refocus the magnetization, but also create longitudinal magnetization. This generates stimulated echoes^16^ that are added to the spin-echo pathway. This generally creates an oscillating behavior of the echo decay curve and overall lengthens the decay, biasing the true T_2_ toward apparent longer T_2_ times if this effect is not considered. The non-uniformity of the B_1_^+^ field across the field of view further exacerbates the aforementioned behavior, which implies that a pure exponential model is not adequate for signal processing.

A fitting procedure that addresses many of the mentioned issues in muscle exploits the extended phase graph (EPG) formalism to model the MESE signal decay with a two-component model: that is intramuscular fat and water. Such an approach is considered the gold-standard to estimate the T_2_ of lean muscle tissue (T_2w_), and it is robust to the presence of fat infiltration^17^. Two main classes of EPG methods exist: nonlinear least square fitting (NLSQ)-based procedures^17^ or dictionary-based procedures^18^. In the NLSQ approach, a non-linear iterative optimization procedure is used to minimize the sum of the squares of the difference between the experimental signal and the EPG-modeled theoretical signal, voxel-wise. In the dictionary-based approach, the experimental MESE signal is matched, voxel-wise, with a precomputed dictionary of simulated signals. Both techniques have been shown to accurately estimate T_2_ of the muscle independently of fat infiltration^17–19^. It should be noted that to obtain accurate T_2w_ the slice profile used in the EPG model should match as close as possible the real slice profile in the MESE acquisition, which requires the knowledge of the pulse shapes used to acquire the data. This should be considered when different vendors apply different proprietary pulses.

Despite their robustness, both methods suffer from high computational burden and long computational time on standard computers, usually requiring several tens of minutes per subject, which limits the wide applicability of these methodologies for research and clinical applications. For the NLSQ procedure, for each pixel several iterations are necessary to find the optimal fit to the experimental data. For the dictionary-based approach, the computational burden arises from the creation of the dictionary and the subsequent exhaustive search across it. Reducing the size of the dictionary by decreasing its resolution is a straightforward strategy to limit the computational burden. However, this may introduce quantization artifacts, as the output MR parameters are limited to those present in the dictionary. The use of high-end computers with the assistance of graphical processing unit (GPU) can accelerate computing^18^, but at the cost of more high-end hardware. Ideally, one would want to maintain the accuracy of EPG fitting procedure while requiring limited computational resources and short processing times.

Long computational times and high computational resources are detrimental for several reasons. To increase fitting accuracy, the T_2_ of the fat component (T_2f_) should be calibrated for each subject applying an EPG fitting procedure in a region of subcutaneous fat. When a large population is studied, to decrease the computational burden associated with the need of computing multiple dictionaries in the dictionary-based approach, T_2_ of the fat component is fixed to a value (estimated from literature or as average of the population under study) and used for all the subsequent muscle T_2_ estimation. However, this procedure can lead to inaccuracy as it has been reported that even small variation in T_2f_ can cause significant differences in muscle T_2_ estimation^19^. Another important motivation for shortening EPG fitting procedures with the use of minimal computational resources is that it could allow real-time processing of the data while a subject is undergoing the MRI exam. This could potentially suggest modifications to the MRI protocol informed by real-time examination of the quantitative T_2_ maps.

A strategy to overcome the computational burden of current EPG fitting methods is to leverage deep learning (DL) algorithms such as neural networks (NN) to predict, voxel-wise, the T_2_ of the lean muscle tissue given the experimental MESE as input after a supervised training procedure. NNs have been demonstrated to be universal function approximators given enough training data and model complexity^20^. A NN can, in principle, learn an approximation of the inverse transfer function that maps the acquired MESE signal into the MR parameter space. Moreover, once the NN is trained, the prediction operation requires extremely limited computational resources. Further, under optimal training conditions, a NN can effectively process signals that were never seen during training. This limits quantization artifacts that can arise from a dictionary approach, in which template matching approximates MR parameters to those present in the dictionary.

The aim of this work was to develop a deep learning approach based on fully connected NN for fast muscle T_2w_ mapping with subject-specific T_2f_ calibration that (i) requires minimal computational resources, and (ii) takes only a few seconds to perform the computation. We validated our method in vivo using the MESE product sequence of two MRI vendors: Siemens and Philips.

The source code to set up and run the application is openly available at https://github.com/barma7/Deep_Learning_for_Muscle_T2_mapping.git.

## Methods

### EPG simulations

The pixel-wise 2D MESE signal was modeled using the EPG algorithm taking into account the effects of slice profiles as described by Lebel and Wilman^21^. A two-component model was used to represent fat and water pools within the muscle tissue according to Eq. 1

1 $$S\left(t\right)=FF\times EPG\left({T}_{1f}, {T}_{2f}, {B}_{1}, TE, EXprofile, REFprofile, ETL\right)+ WF\times EPG\left({T}_{1w}, {T}_{2w}, {B}_{1}, TE, EXprofile, REFprofile, ETL\right)$$

where *T*_*1f.*_*, T*_*2f.*_*, T*_*1w*_ and *T*_*2w*_ are the fat and water longitudinal and transverse relaxation times, respectively; *B*_*1*_ is the pulse efficiency ratio; *TE* is the echo time space; *EXprofile* and *REFprofile* are the excitation and refocusing slice profiles, respectively; *ETL* is the echo train length (number of refocusing pulses in the MESE sequence); *FF* and *WF* are the fat and water fractions, respectively.

T_1f._ and T_1w_ were fixed to 365 and 1400 ms, respectively^17^. Given the excitation and refocusing pulse shapes of Philips and Siemens MESE sequences used, the slice profiles were obtained using the Shinnar-Le Roux (SLR) algorithm^22^. All simulations were performed using an in-house MATLAB (MathWorks, release R2022b) library based on the StimFit toolbox^23^.

### Deep learning approach: NN model and training pipeline

**Figure 1:**
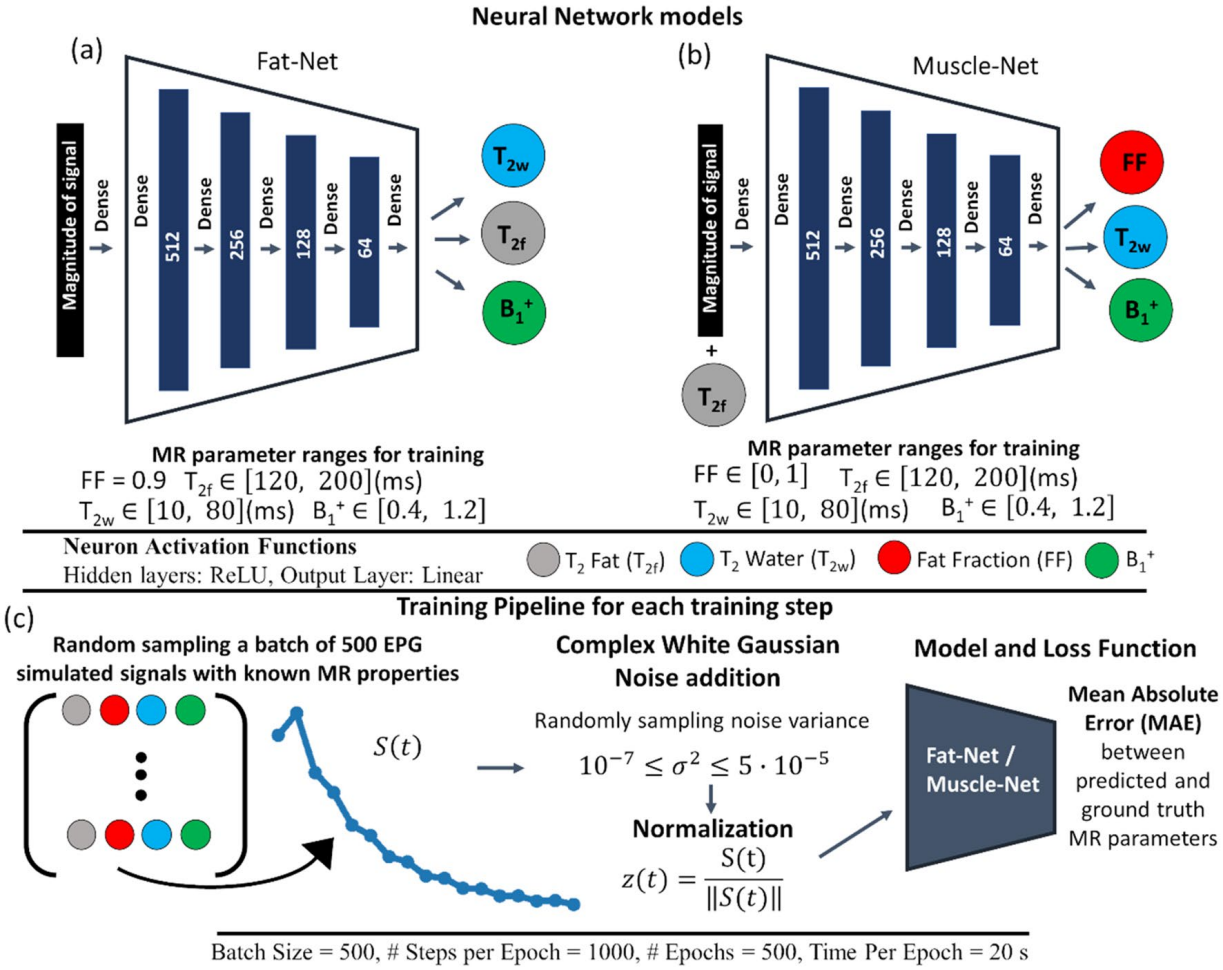
Schematization of the NN models and the training pipeline used for training. (**A**) Fat-Net and (**B**) Muscle-Net have the same architecture: 4 fully connected hidden layers with a progressively decreasing number of neurons. (**C**) During training, simulated data are augmented by injecting white gaussian noise with different noise variances. Signals are normalized before being fed to the NN.

The Neural Network application was developed in Python using Keras with TensorFlow^24^ backend and is summarized in Fig. 1. Two NN models for pixel-wise processing were defined: Fat-Net (Fig. 1a) and Muscle-Net (Fig. 1b). Fat-Net estimates T_2f_, T_2w_ and B_1_^+^ taking the MESE signals coming from regions of sub-cutaneous fat as input. Muscle-Net predicts the fat-fraction (FF), T_2_ of the lean muscle component (T_2w_) and B_1_ given the MESE signal and an estimate of T_2f_. as inputs. Both models have 6 fully connected layers, the Rectified Linear Unit (ReLU) was used as the activation function for the neurons in the first 5 hidden layers, while a linear activation function was used for the output layer. A bottle-neck structure was chosen as it has been successfully applied in other quantitative MR applications^25,26^.

Each model was trained using MESE data simulated using the EPG formalism with a supervised procedure, with the mean absolute error (MAE) between NN estimated parameters and ground-truth parameters as loss function. The Adam algorithm^27^ was used as optimizer using 500 training epochs with 1000 gradient steps for each epoch, with a fixed batch size of 500. The training sets were composed of 100,000 MESE signals generated by randomly sampling the parameter space according to a uniform distribution. Specifically, for Fat-Net, subcutaneous fat signals were generated by randomly sampling T_2f._ and T_2w_ in the ranges [50, 250] ms and [10, 110] ms, respectively, using a fixed FF value of 0.9. For Muscle-Net, muscle tissue signals were generated by randomly sampling T_2f._, T_2w_ and FF in ranges: [50, 250] ms, [10, 80] ms and [0, 1] respectively. For both training sets, B_1_^+^ was sampled within the range [0.4, 1.2].

With reference to Fig. 1c, to promote model generalization and robustness to noise, for each training step, simulated noiseless MESE signals were corrupted by white Gaussian noise with different variances, which are randomly sampled from a uniform distribution with ${10}^{-7}\le {\sigma }^{2}\le 5\cdot {10}^{-5}$. Using a wide range of noise variances has been proved to be the best noise addition strategy when a similar NN architecture was used for MR pixel-wise parameter mapping^26^. For training Fat-Net a narrower range of noise SNR (${10}^{3}\le SNR\le {10}^{4}$) was used as pixels in the subcutaneous fat displays higher SNR than pixels in the muscles. Preliminary tests empirically showed that reducing the noise range to higher SNR values improved T_2f._ estimation. After the data augmentation step, the magnitude of each input was taken and normalized to the unit norm. When applied to real acquired data for pixel-wise processing, the experimental MESE data is first normalized to the unit norm, by dividing the signal with its norm, and then fed to the pre-trained network model.

Because the product MESE sequence of the two vendors utilized different pulse shapes, two deep learning applications were developed independently for Siemens and Philips by using training data simulated with their corresponding proprietary pulses.

### Reference EPG fitting procedures

Two EPG fitting procedures conventionally adopted in the literature were used as reference against the proposed DL application. They were NLSQ^17^ and dictionary matching^18^ (DM) fitting. For the NLSQ, the MR parameter spaces for fat calibration and muscle fitting were constrained within the same ranges used for training Fat-Net and Muscle-Net, respectively. For the Dictionary application, two dictionaries were created for fat calibration and muscle processing, respectively. Specifically, for fat calibration FF was fixed to 0.9 and T_2f._, T_2w_ and B_1_^+^ values spanned the ranges [50, 250] ms, [10, 110] ms and [0.4, 1.2], respectively, with steps sizes of 0.25 ms, 0.5 ms and 0.025 ms, respectively. For muscle processing, T_2f._ was fixed to the calibrated value while FF, T_2w_ and B_1_^+^ spanned the ranges [0, 1], [10, 80] ms and [0.4, 1.2], respectively, with steps sizes of 0.015, 0.2 and 0.025 ms, respectively. Both applications were developed in MATLAB R2022b and were optimized for computational efficiency through code vectorization and exploitation of MATLAB’s automatic parallel support for multicore CPU. As for the deep learning approach, different dictionaries were created for Siemens and Philips by using their corresponding proprietary pulses used in the MESE sequence. Similarly, for the NLSQ fitting procedure the corresponding proprietary pulses were used for EPG modelling during least square minimization when analyzing Siemens and Philips data.

### Computer configuration and computational time

All applications (deep learning, dictionary and NLSQ) were run on a standard laptop equipped with 16 Gb of Random Access Memory (RAM) using only Central Processing Units (CPUs): Intel Core i7-8565U. The Graphical Process Unit (GPU) integrated in the laptop, an Intel UHD Graphics 620, was not used at any point. With such configuration, each deep learning model required 20 s per epoch, resulting in a total of 2 h and 42 min to complete the training. For the dictionary approach, 250 s were needed to create the dictionary for fat calibration, whereas the dictionary for muscle T_2w_ estimation required 1.2 s once T_2f._ was calibrated.

### MRI acquisitions

A multi-stack upper leg bilateral scan of ten healthy subjects was performed at 3 T. Five were scanned using a Philip scanner (Ingenia, Philips, Best, Netherlands) for an unrelated study^28^, and five were scanned using a Siemens scanner (MAGNETOM Skyra, Siemens Healthineers, Erlangen, Germany). The MRI protocol included a Dixon water/fat-separated sequence for anatomical reference and for fat fraction estimation, and a MESE sequence for water T_2_ mapping. Sequence parameters for each scanner are reported in Table 1. The variation in acquisition parameters between Siemens and Philips scanners reflects the default settings that each vendor set to optimize acquisition time and SNR given a specific product sequence. In this work, our goal was to use product sequences optimized by the vendor for the specific parameter of interest. Thus, the variation in acquisition parameters between Siemens and Philips scanners reflects the settings that each vendor set for optimizing acquisition time and SNR given a specific product sequence. For the MESE sequence, the focus of our analysis, the main difference resides in the TR (5633 vs 2870 ms for Siemens and Philips, respectively). TR needs to be long enough to allow full recovery of the longitudinal magnetization to its equilibrium value to minimize T_1_-weighting. To achieve such a goal, TR is normally set to a value between 2 and 4 times the longest T_1_ of the component of interest. In our case T_1_ of the water component is about 1400 ms while T_1_ of the fat component is about 365 ms. TR for both vendors were long enough to minimize T_1_-weighting and it therefore unlikely that this difference in TR could significantly affect T_2_ analysis.

**Table 1:** Sequence parameters used for the MRI protocols run using Philips and Simens scanners.

Scanner	PHILIPS		SIEMENS	
Sequence type	T_2_ mapping	DIXON	T_2_ mapping	DIXON
Sequence name	MESE	MS-FFE	MESE	GRE-ME
Matrix size	160 × 160	320 × 320	132 × 192	172 × 288
Voxel size (mm^3^)	3 × 3 × 6	1.5 × 1.5 × 6	2 × 2 × 6	1.4 × 1.4 × 5
Slice gap (mm)	6	–	6	–
Slices	17	31	15	52
TR (ms)	5633	210	2870	17.15
TE (ms)	7.6	2.6/3.36/4.12/4.88	7.5	1.3
ETL	17	4	17	12
Flip Angle	90º/180º	8º	90º/180º	9º
Acquisition Time (m:s)	4:32	1:25	5:55	0:21

*FFE* Fast Field Echo, *GRE* Gradient Recalled Echo, *ME* Multi-Echo, *MESE* Multi-Echo Spin-Echo, *MS* Multi-Slice.

### Data processing and analysis

**Figure 2:**
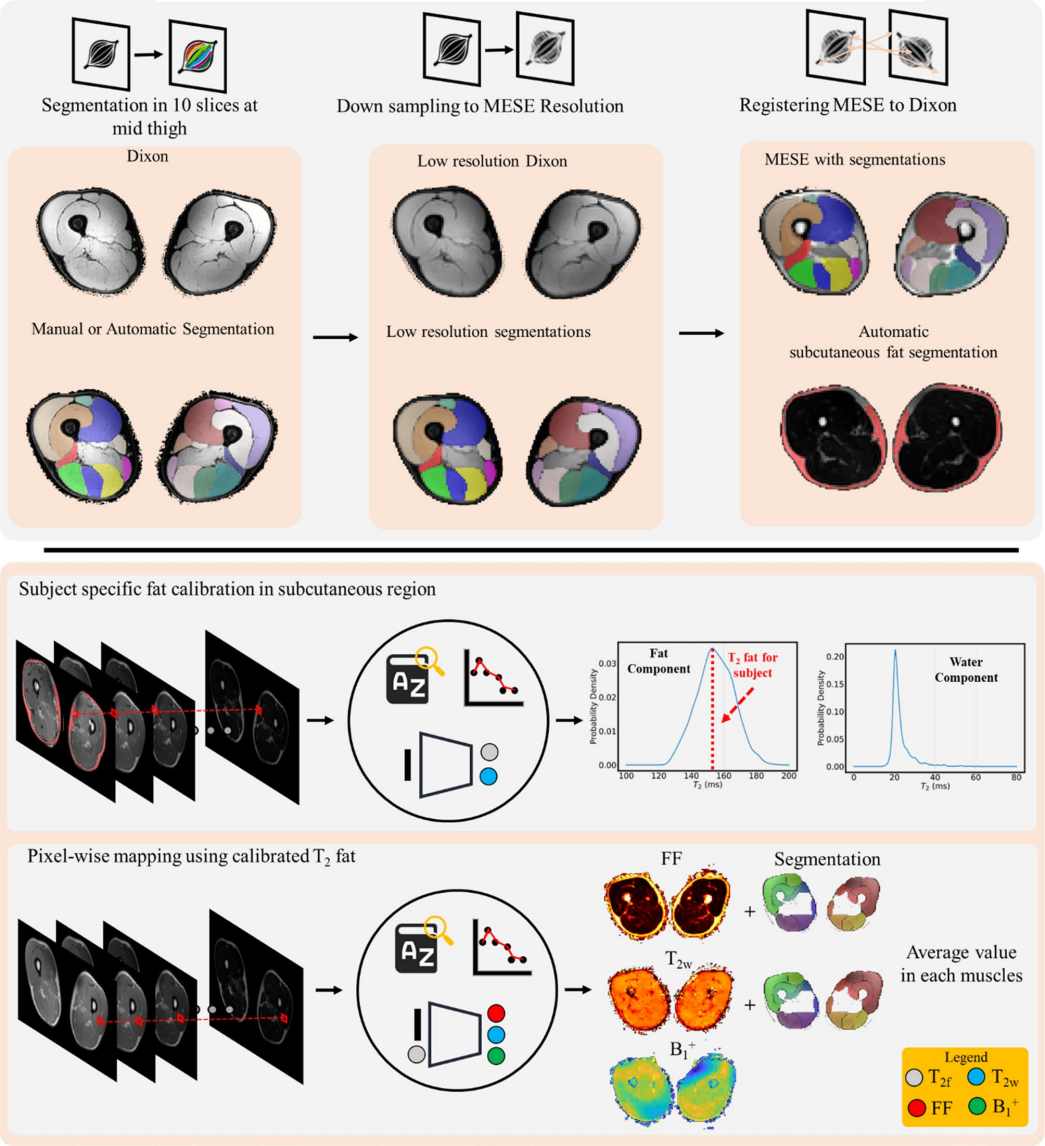
Processing pipeline used to analyze the data. Image pre-processing (top panel) and data analysis (bottom panel) steps used to produce the parametric maps for each subject: T_2w_, FF and B_1_^+^ with subject specific T_2f._ calibration.

The data pre-processing pipeline is summarized in top panel of Fig. 2. For each subject, ten muscles (Vastus Medialis, Vastus Lateralis, Vastus Intermedius, Rectus Femoris, Biceps Femoris Long Head, Biceps Femoris Short Head, Semimembranosus, Semitendinosus, Sartorius and Gracilis) in both legs were segmented in 10 slices of the out-of-phase Dixon images acquired at mid-thigh. For Siemens data, segmentation was carried out automatically using the Deep Anatomical Federated Network (DAFNE) collaborative platform^29^ and subsequently manually verified and corrected to ensure accuracy of the segmentation. For Philips data, muscles were manually segmented. For both vendors and for each subject, the Dixon images were then rescaled to match the MESE resolution and registered to the first MESE echo using Elastix^30^. Lastly, a region of subcutaneous fat was selected with an automatic K-means clustering segmentation based on the Lloyd’s algorithm^31^ using the first echo of the MESE echo. Subcutaneous segmentations were also manually checked and corrected if necessary to avoid inclusion of background or muscle pixels within the subcutaneous mask.

Once the data underwent the pre-processing pipeline described above, quantitative analysis was performed as summarized in the bottom panel of Fig. 2. For each vendor and for each subject, to obtain T_2w_ of the muscle component, T_2f._ was first calibrated in the subcutaneous region using the pre-trained Fat-Net, and fed as input to the pre-trained Muscle-Net. The estimation was also repeated using both dictionaries matching and NLSQ fitting procedures for reference. The average T_2w_ value in each muscle was calculated. We used Bland-Altmann analysis^32^ to compare the T_2w_ values obtained with our deep learning approach to the values obtained using the reference fitting methods. The limits of agreement (LOA) and the Lin’s concordance correlation coefficient^33^ (r_c_) were used as global metrics of agreement between methods A separate analysis was conducted for Siemens and Philips data as the MESE sequence parameters and the pulse shapes used differed between the two vendors. Furthermore, the reference average FF in each muscle was computed from the Dixon FF maps, which were obtained, pixel-wise, using Eq. 2, where *I*_*w*_* and I*_*f*_ are the water-only and fat-only images output by the scanner. For each T_2w_ fitting method, a BA analysis comparing the reference Dixon-based FF values with the FF estimated by the bi-component fit was performed. Lastly, the correlation between average T_2w_ values and the Dixon-based FF values was also assessed.

2 $$FF=\frac{{I}_{f}}{{I}_{w}+ {I}_{f}}\times 100 (\%)$$

## Results

**Figure 3:**
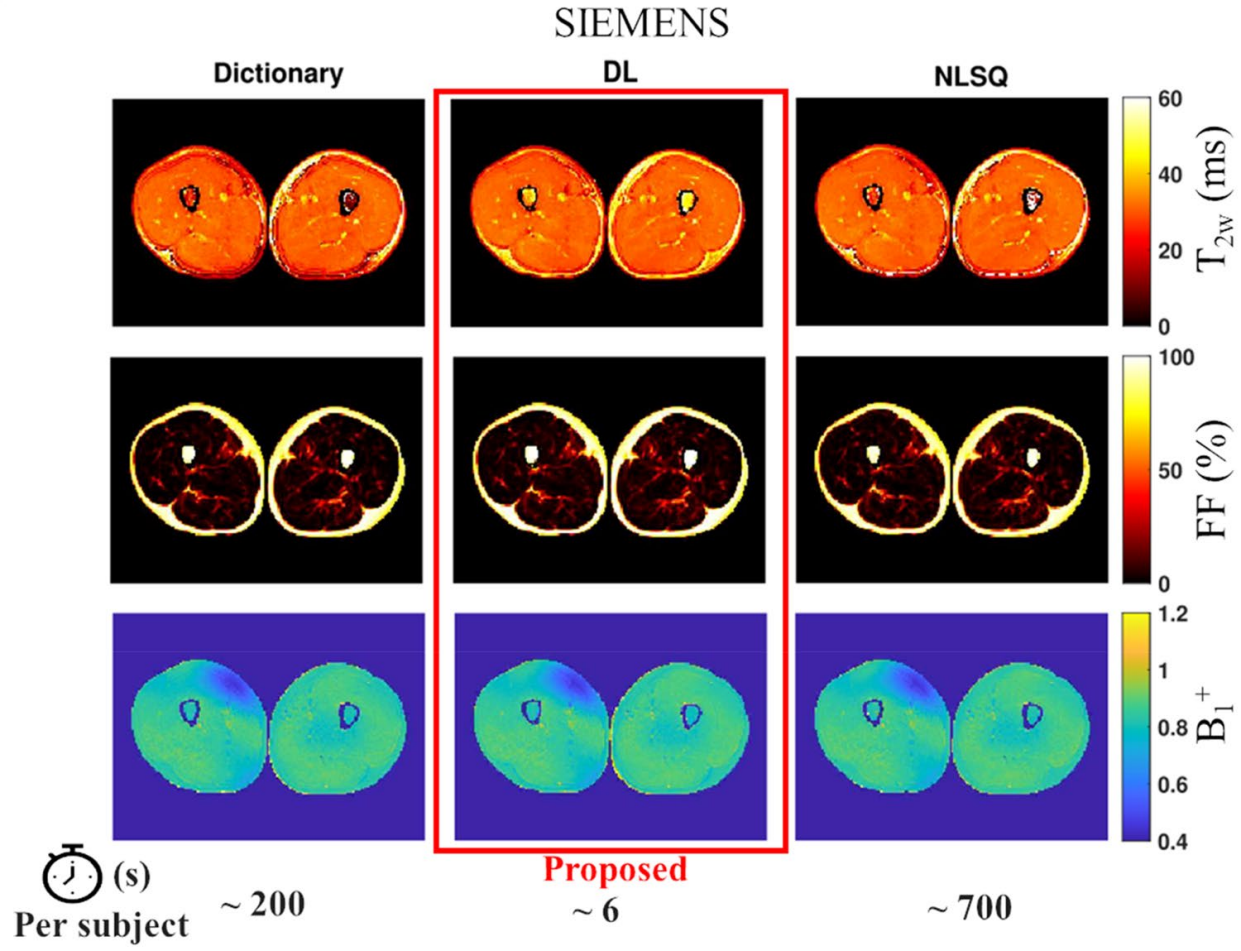
Example of T_2w_, FF and B_1_^+^ quantitative maps obtained in the thigh from EPG fitting of MESE data acquired with Siemens scanner. The proposed deep learning approach (central column, red frame) is presented along with the maps obtained using the dictionary (left column) and NLSQ (right column) reference fitting methods.

**Figure 4:**
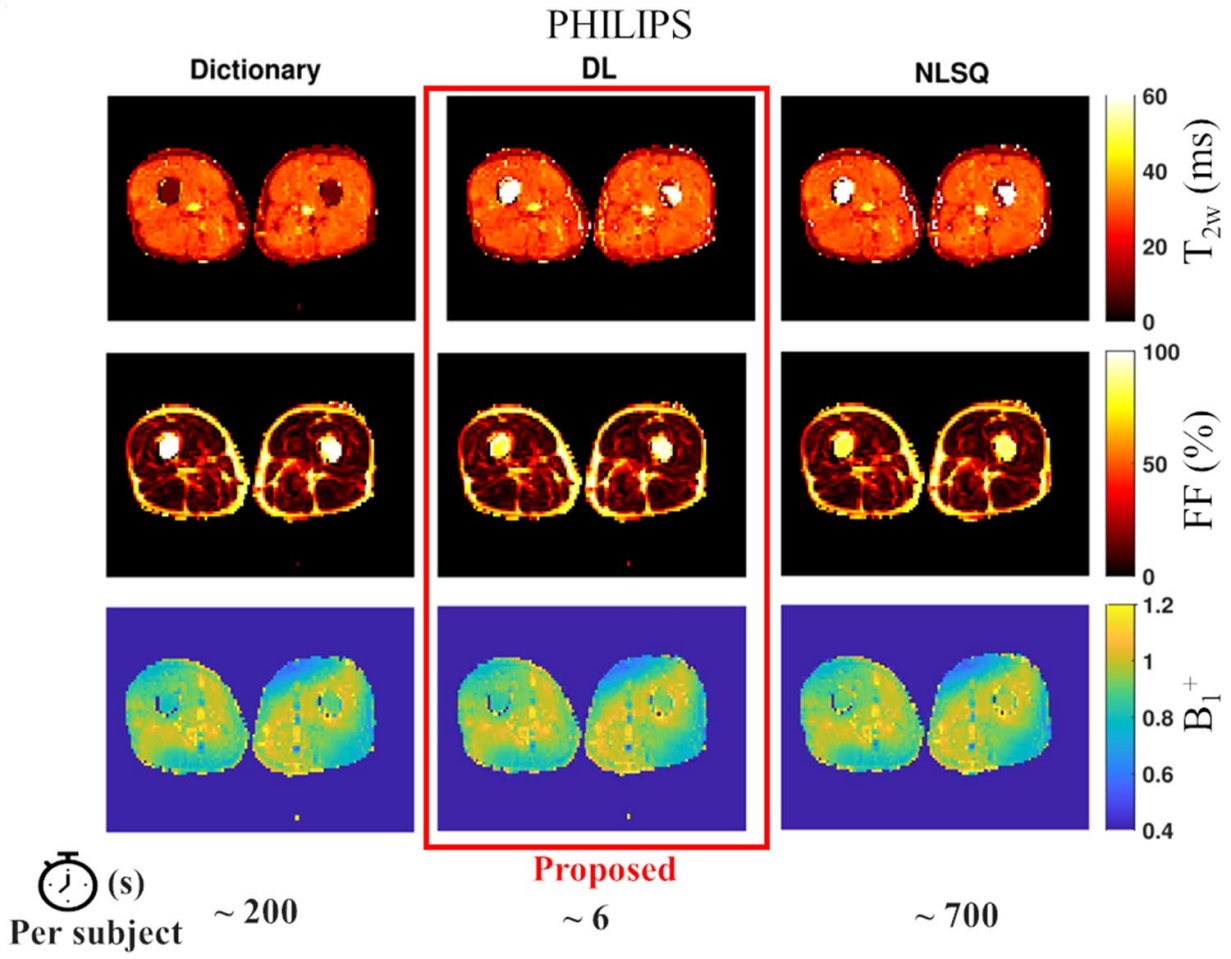
Example of T_2w_, FF and B_1_^+^ quantitative maps obtained in the thigh from EPG fitting of MESE data acquired with Philips scanners. The proposed deep learning approach (central column, red frame) is presented along with the maps obtained using the dictionary (left column) and NLSQ (right column) reference fitting methods.

Representative parameter maps reconstructed using the proposed DL approach and the reference methods for Siemens and Philips dataset are shown in Figs. 3 and 4, respectively. For both datasets the quantitative maps appear to be similar across fitting methods by visual inspection and no artefacts are noted. The average computational time required to perform fat calibration and process all the slices in a subject were 6 s, 200 s and 700 s for the DL, dictionary and NLSQ methods, respectively.

**Figure 5:**
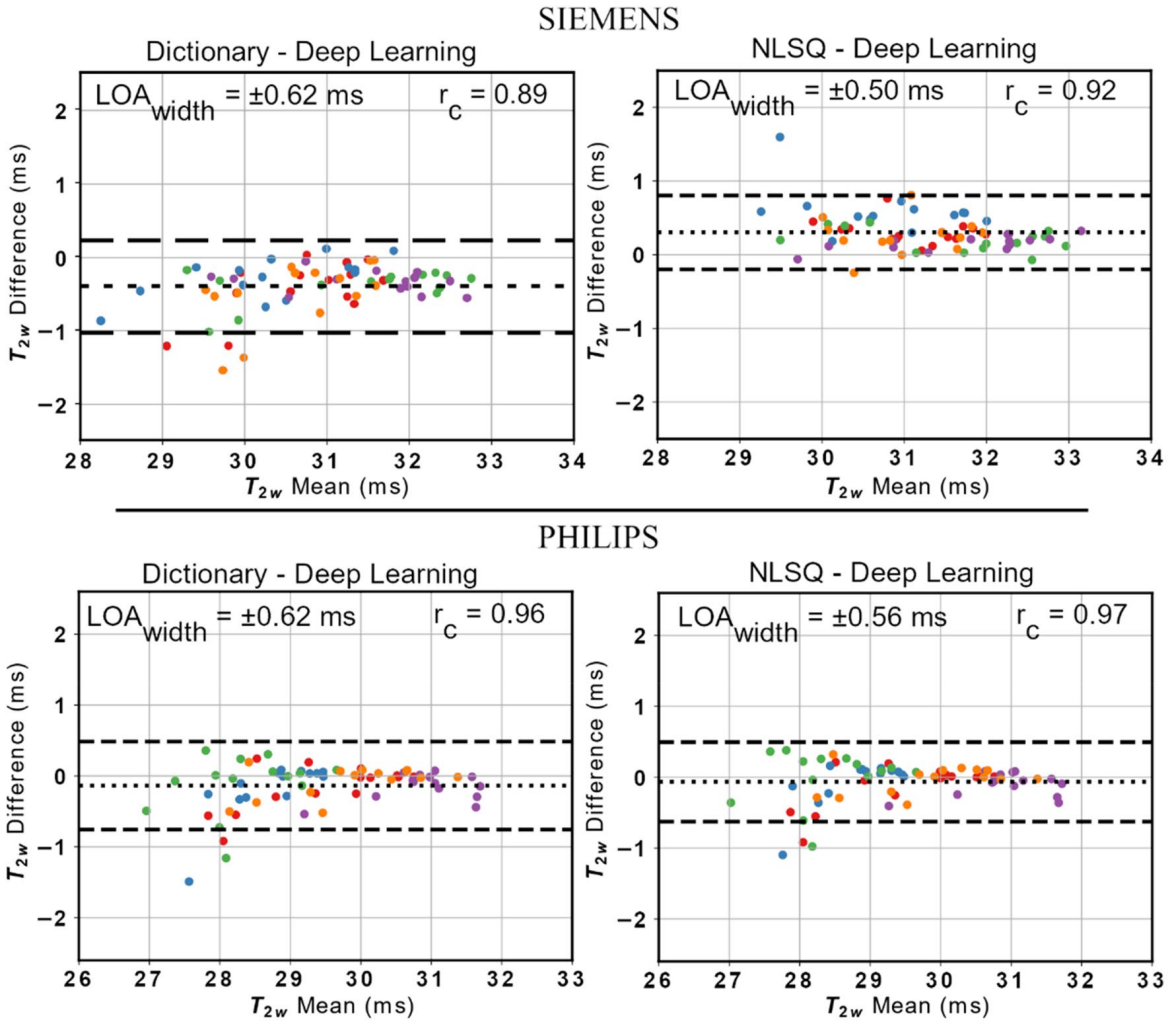
Bland–Altman plots reporting the comparison of T_2w_ values obtained using the prosed DL approach with the reference dictionary (left) and NLSQ (right) EPG fitting methods for data acquired using Simens (top panel) and Philips (bottom panel) scanners. The width of the LOA is reported for each BA plot along with the Lin’s concordance coefficient. The different colors represent muscle T_2w_ values belonging to different subjects.

**Figure 6:**
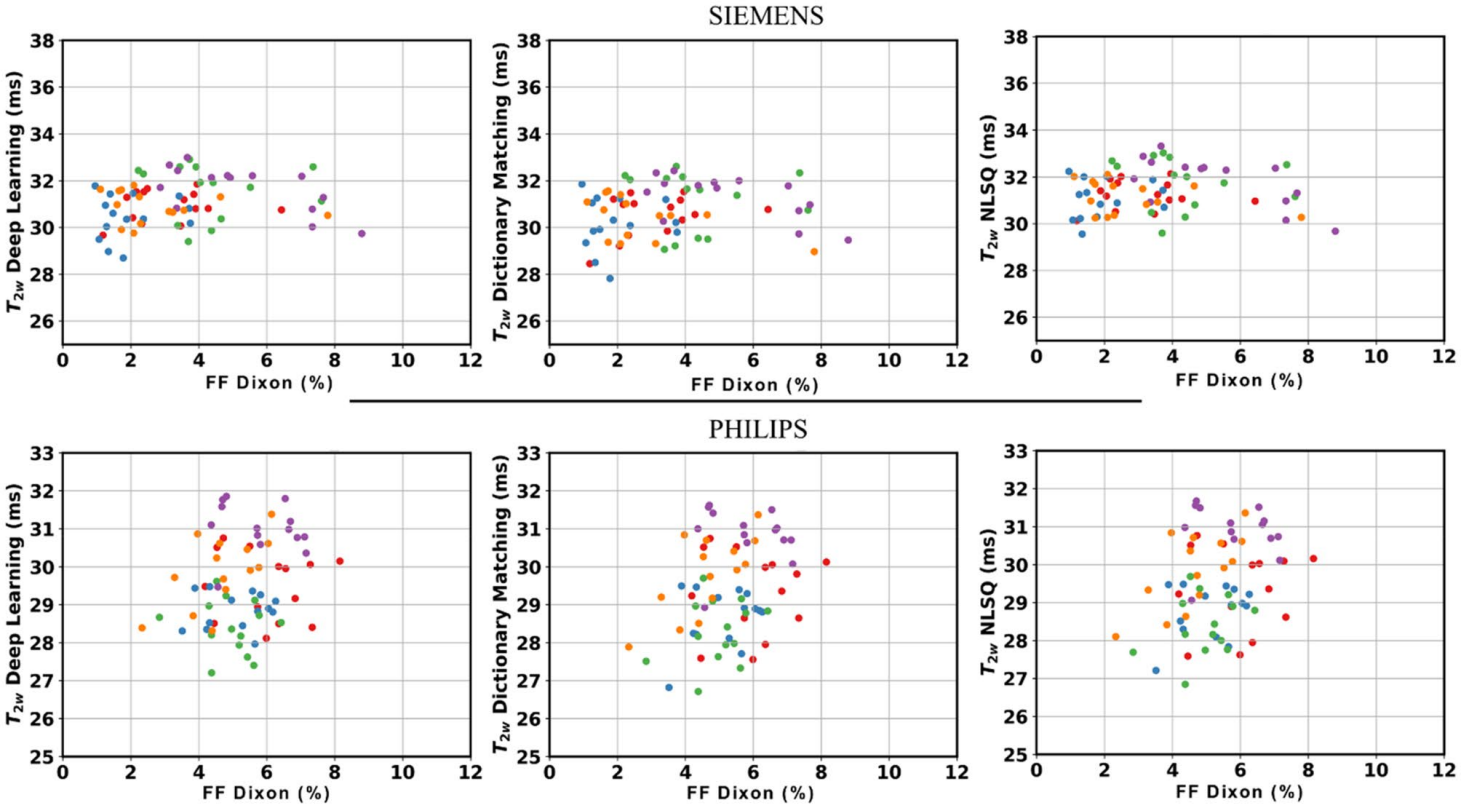
Scatter plots of estimated T_2w_ values against Dixon fat fraction values. The plots are reported separately for Siemens (top row) and Philips data (bottom row). A plot for each EPG fitting procedure investigated is reported: the proposed DL approach and the reference dictionary and NLSQ methods. The different colors represent values belonging to different subjects.

The BA plots for each scanner are presented in Fig. 5. The T_2w_ values estimated using the proposed DL approach were in very good agreement with those obtained with the dictionary approach based on dot-product metric and with those computed using the NLSQ fitting procedure. Specifically, in Siemens data when comparing the DL approach with the dictionary and NLSQ methods, respectively, r_c_ were equal to 0.89 and 0.92, respectively, and the widths of LOA were equal to ± 0.62 ms and ± 0.50 ms, respectively. Similarly, when looking at Philips data, r_c_ were equal to 0.96 and 0.97, respectively, and the widths of LOA were equal to ± 0.62 ms and ± 0.56 ms, respectively. In Siemens, small, but statistically significant, biases equal to − 0.4 ms and 0.3 ms were found between T_2w_ values evaluated using the DL approach and T_2w_ values obtained with the dictionary and the NLSQ reference methods, respectively. In Philips, only a small bias equal to − 0.1 ms between T_2w_ values evaluated using the DL approach and T_2w_ values obtained with the reference dictionary method was statistically significant. Similarly, very good agreement was found between B_1_^+^_,_ and FF values estimated using the proposed DL approach and the reference methods, as reported in the BA plots in Figures S1 and S2. Concerning FF accuracy, the BA plots comparing the FF values estimated using the EPG methods and the reference FF values from Dixon are reported in Figure S3. The EPG method overestimated the reference FF by about 3 percentage points (p.p.) regardless of the fitting procedure utilized. Further, a positive linear relationship between FF deviation and FF value was also observed. However, this was independent of the fitting method used (NLSQ, DM or NN). Finally, as highlighted in Fig. 6, no statistically significant correlation was found between the reference FF values obtained with Dixon and T_2w_ values obtained used the 3 EPG methods, indicating that they can successfully remove the contribution of fat from the MESE signal.

## Discussion

In this work, we developed a neural network approach for fast muscle T_2w_ mapping with subject-specific T_2f._ calibration to overcome the computational burden and the lack of flexibility of current bi-component EPG fitting methods. This method was validated in vivo using the MESE product sequence of two MRI vendors, Siemens and Philips.

Our DL approach was able to predict T_2w_ values that were in strong agreement with those obtained using the reference NLSQ and dictionary approaches, while reaching a 116 × and 33 × computational time improvement, respectively. We observed a sub-millisecond bias in the T_2W_ negative obtained with our method compared to NLSQ and dictionary matching. However, these biases were smaller than the bias observed between these two reference methods. A bias of 0.9 ms between the dictionary matching method and the NLSQ methods has been previously reported in literature^18^ and it was consistent with the bias observed in this work. Furthermore, we observed that inter-method biases in the Siemens dataset were overall higher than the biases observed in the Philips dataset. Although we did not further explore the motivation of such difference, this could be related to the fact that, on average, B_1_^+^ efficiency observed in the Siemens acquisitions was about 15% less efficient than the one measured in Philips acquisitions. The EPG method accounts for B_1_^+^ inhomogeneity, but this could still have an impact as fitting metrics and optimization methods differ among the approaches. We observed that the EPG method overestimated the FF of about 3 p.p compared to the reference Dixon values and observed a positive relationship between FF bias and FF value. Although this is not of primary interest or concern, it is worth commenting on what could lead to such bias and behavior. Among several factors that might contribute to it, a major one could lie in the difference between the EPG-based and Dixon-based methods for water-fat separation. Dixon imaging is based on the chemical shift between water and fat. The EPG method does not consider the chemical shift between water and fat, as it models data acquired with MESE sequences. However, it has been recently shown that accounting for chemical shift artefacts in the slice direction due to misalignment of the slice profiles between water and fat improves accuracy of FF estimation in EPG fitting of MESE data^19^. Thus, such artefacts could be included in the modelling for improved FF accuracy. Nonetheless, the deviation between EPG-based and Dixon-based FF estimations did not adversely impact the obtained results in terms of muscle T_2w_ estimation, as no statistically significant correlation between T_2w_ values and reference Dixon-based FF was observed. This suggests that each EPG method successfully separated the T_2w_ from fat. For the NLSQ and dictionary method this behavior has been already observed^18^. The observation that also the proposed DL approach showed such behavior increases confidence on the method. However, it should be noted that only young healthy subjects were included in the current study. Thus, although we do not expect our DL approach to behave differently from the reference EPG methods, the absence of a positive correlation between T_2w_ and FF values should be verified when the method is applied to older subjects and patients with neuromuscular diseases, as a much higher FF is to be expected in this case.

It is worth noting that regardless of the method used, estimation of muscle T_2w_ in region where FF is close to 1 is not reliable. In such regions (for example bone marrow and subcutaneous fat), the amplitude of the water component is close to zero leading to very low SNR of the water signal compared to the SNR of the fat signal. In such cases, the fit tends to predict T_2w_ values equal to (or close to) either the lower or higher bound set for the fitting regardless of the method used. The metric and optimization algorithm used to perform the fitting influences whether the lower or higher bound is more likely to be selected. In our work all three methods are different in terms of metric and optimization algorithm used. Thus, it is expected to have different behaviors in regions where the water component has very low SNR compared to the fat component. As a result, the differences in T_2__w_ estimation in the bone marrow region among methods observed in Figs. 3 and 4 are of no concern.

The most relevant benefit of our approach lies in the high computational efficiency and fast processing times (6 s per subject) to obtain muscle T_2w_ mapping with subject specific T_2f._ calibration. Even small variations in T_2f._ have been shown to cause differences up to 4 ms in T_2w_^19^, and we observed a relatively wide range of T_2f._ values in a cohort of healthy participants as reported in Figure S4, which is expected to be even wider in patients. However, in practice often a single fixed T_2f._ is assumed for dictionary matching approaches to keep storage and computational resources manageable, resulting in a bias in T_2w_ estimation. The approach can be speeded up with the use of parallel computing paired with Graphical Process Units (GPUs), which is the strategy adopted by the open-source toolbox MyoQMRI^18^. In the paper explaining the toolbox, Santini et al. reported an average processing time of 2 s/slice, which if applied to our case would have led to a total computational time of about 30 s per subject. This would be 6.6 × faster than our CPU-based dictionary-matching approach, and about 5 × slower than the proposed CPU-based deep learning approach. Furthermore, with increased dictionary size, more memory on the device is needed to exploit benefit from GPU parallel computation. Depending on the application, one may want to increase the resolution of the dictionary, and the higher required memory eventually becomes the limiting factor despite the use of GPUs in dictionary-based approaches. With the proposed DL approach this task is easily handled even with limited computational resources. For example, execution of our application was performed on a middle-range laptop equipped with 16 Gb of RAM and used only CPUs power (Intel Core i7-8565U). Another approach to increase computational efficiency of the dictionary matching approach is to use a low-rank approximation of the dictionary using singular value decomposition (SVD). In the case of MESE, three principal components allow to retain more than 99% of the total variance present within the dictionary as observed in Figure S5. We observed a 15% reduction in computational time (from about 200 s to about 175 s for a single subject) when using SVD compression compared to the “conventional” dictionary matching approach. It is worth pointing out that we did not systematically evaluate the effect of SVD compression on T_2f._ and T_2w_ estimations when comparing it with the un-compressed dictionary method. Since more than 99% of the total variance is retained with 3 components, one should expect the deviation with the non-SVD compressed case to be negligible. However, such deviation should be quantitatively characterized before using SVD compression for muscle T_2w_ analysis. Notably, our NN method outperforms the dictionary-matching methods in terms of computation time, even when SVD compression is used.

One limitation of our approach is that a new training is required whenever MESE sequence parameters (TE, ETL, pulses) are changed. However, it is common to use only one MRI protocol in clinical and research studies. In such cases, fast processing with our DL approach outweighs the time required to train the NN models, which with our configuration required little less than 3 h. Such time can be significantly reduced with the use of GPUs. Thus, one could train the model on a high-end computer and exploit the benefits of DL processing with minimal required computational resources: potentially the processing could be directly performed at the scanner, facilitating implementation into clinical practice as no advanced post-processing would be needed. Another potential limitation is that estimation of T_2w_ values is sensitive to the selection of the slice-profile. Thus, it is important to obtain detailed information about the timing of the MESE sequences and the shapes of the excitation and refocusing pulses, as using? incorrect pulse profiles may result in biases in T_2w_ estimation. However, it is worth mentioning that this limitation is inherent with any EPG fitting algorithm and not exclusive to our method.

In conclusion, we developed a DL approach for fast muscle T_2w_ mapping with subject specific T_2f._ calibration that outperformed cutting-edge literature methods in required computational time and resources while providing same quantitative accuracy. This methodology allows the processing of T_2w_ data in a few seconds, compared to tens of minutes required with current methods, and will facilitate the use of quantitative T_2w_ maps of muscle in clinical and research studies.

### Supplementary Information

Supplementary Figures.

## Data Availability

The source code to develop and run the deep learning application, as long as the conventional EPG fitting methods is available at https://github.com/barma7/Deep_Learning_for_Muscle_T2_mapping.git. The MRI data and source code to run the data analysis to reproduce the findings of this study are openly available in *Zeonodo* at https://doi.org/10.5281/zenodo.10520542.
