# Supplementary material for: A deep learning approach for fast muscle water T2 mapping with subject specific fat T2 calibration from multi-spin-echo acquisitions
Source: Sci Rep. 2024 Apr 8;14:8253. doi: 10.1038/s41598-024-58812-2 (PMC11002020; doi:10.1038/s41598-024-58812-2)
Supplement: Supplementary file 1 — Supplementary Figures. [file 41598_2024_58812_MOESM1_ESM.docx]

SUPPLEMENTARY INFORMATION FOR:

A Deep Learning Approach for Fast Muscle Water T2 Mapping with Subject Specific Fat T2 Calibration from Multi-Spin-Echo Acquisitions

Marco Barbieri^1^, Melissa T. Hooijmans^2^, Kevin Moulin^3^, Tyler E. Cork^1^, Daniel B. Ennis^1^, Garry E. Gold^1,4^, Feliks Kogan^1^ and Valentina Mazzoli^1,5^

^1^ Department of Radiology, Stanford University, Stanford, CA, USA

^2^ Department of Radiology and Nuclear Medicine, Amsterdam University Medical Center, Amsterdam, the Netherlands

^3^ Department of Cardiology, Boston Children’s Hospital, Harvard Medical School, Boston, Massachusetts, USA.

^4^ Department of Bioengineering, Stanford University, Stanford, CA, USA

^5^ Bernard and Irene Schwartz Center for Biomedical Imaging, Department of Radiology, New York University School of Medicine, New York, NY, USA


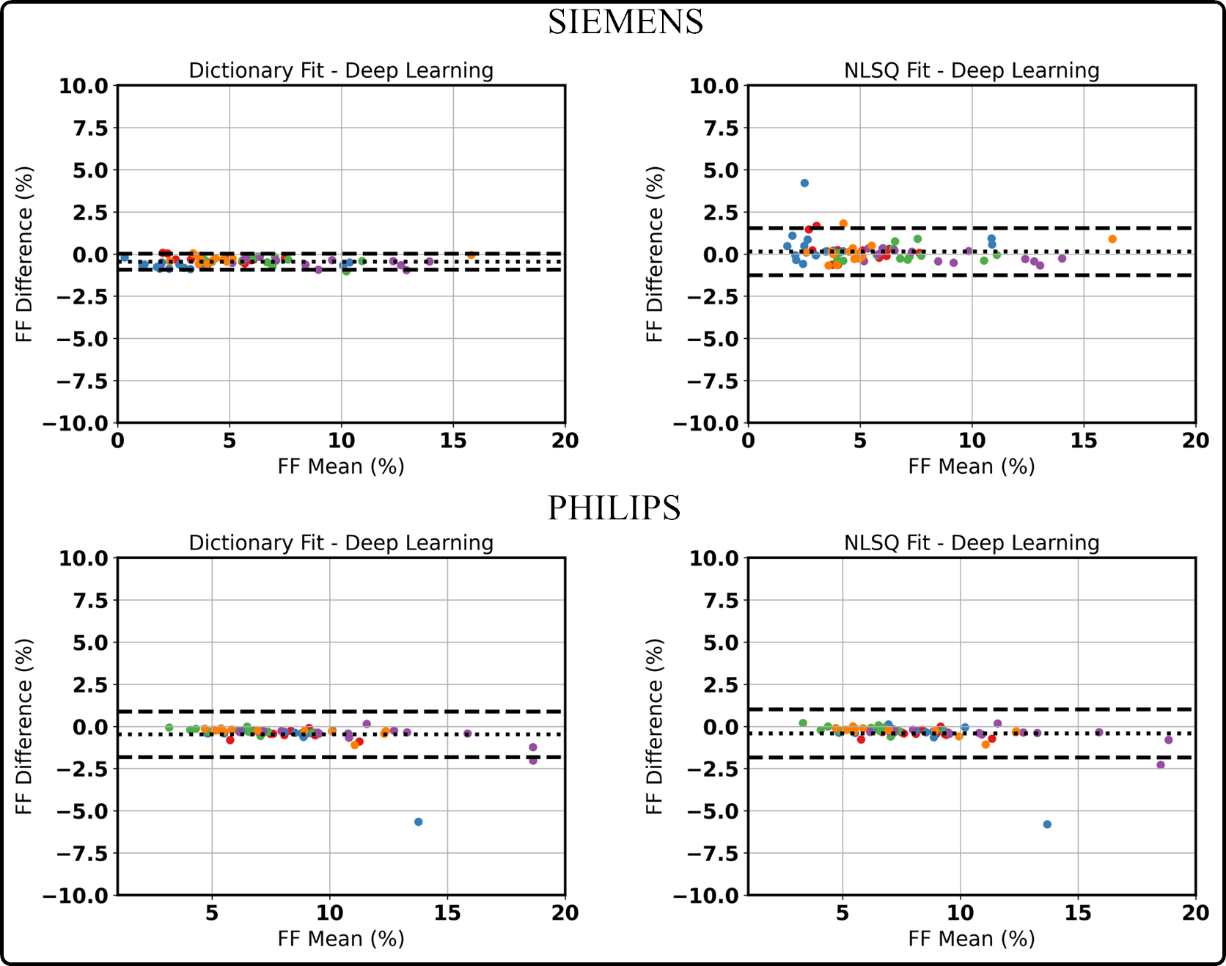


**Figure S1** Bland-Altman plots reporting the comparison of FF values obtained using the prosed DL approach with the reference dictionary (left) and NLSQ (right) EPG fitting methods for data acquired using Simens (top panel) and Philips (bottom panel) scanners.


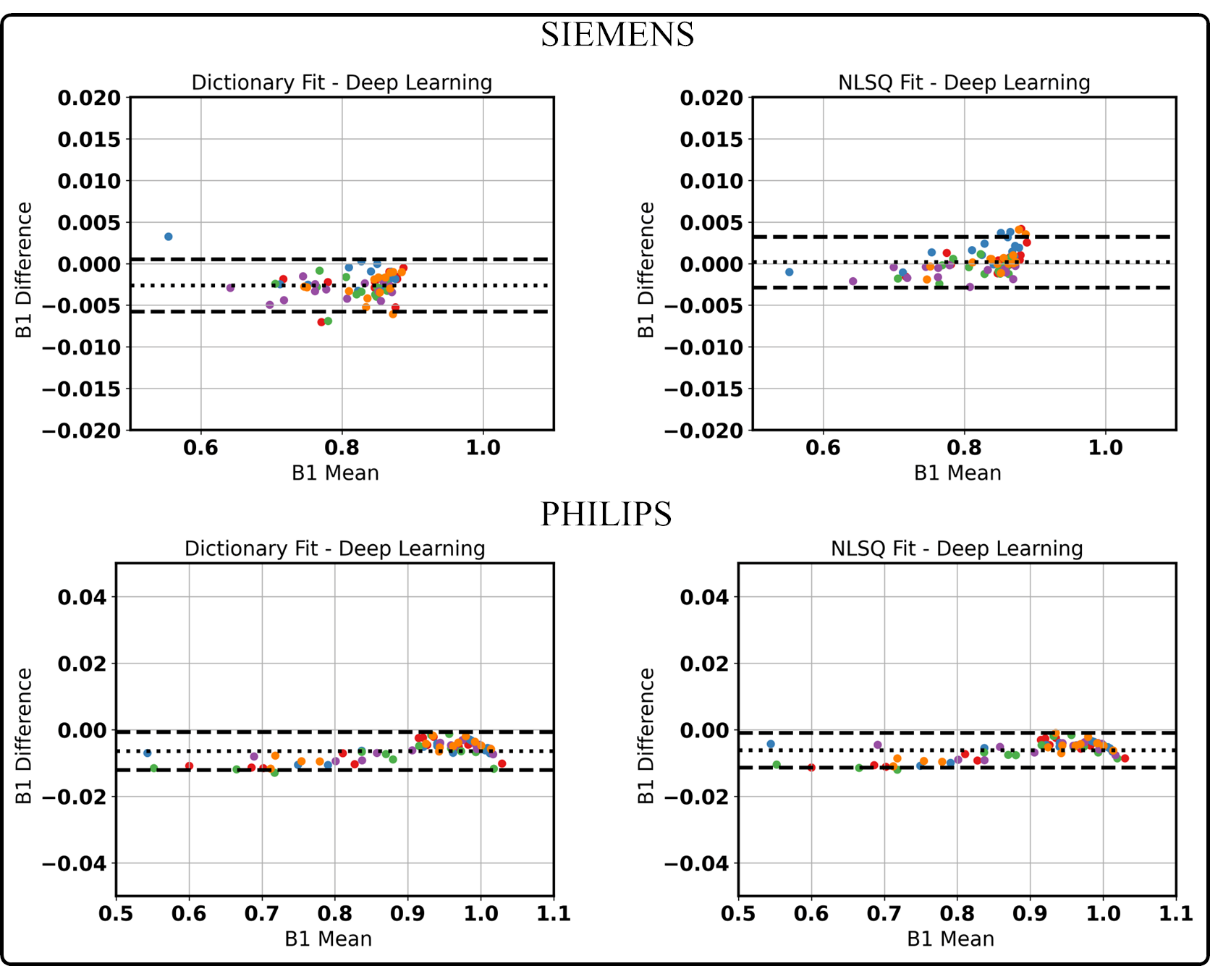


**Figure S2** Bland-Altman plots reporting the comparison of B_1_^+^ values obtained using the prosed DL approach with the reference dictionary (left) and NLSQ (right) EPG fitting methods for data acquired using Simens (top panel) and Philips (bottom panel) scanners.


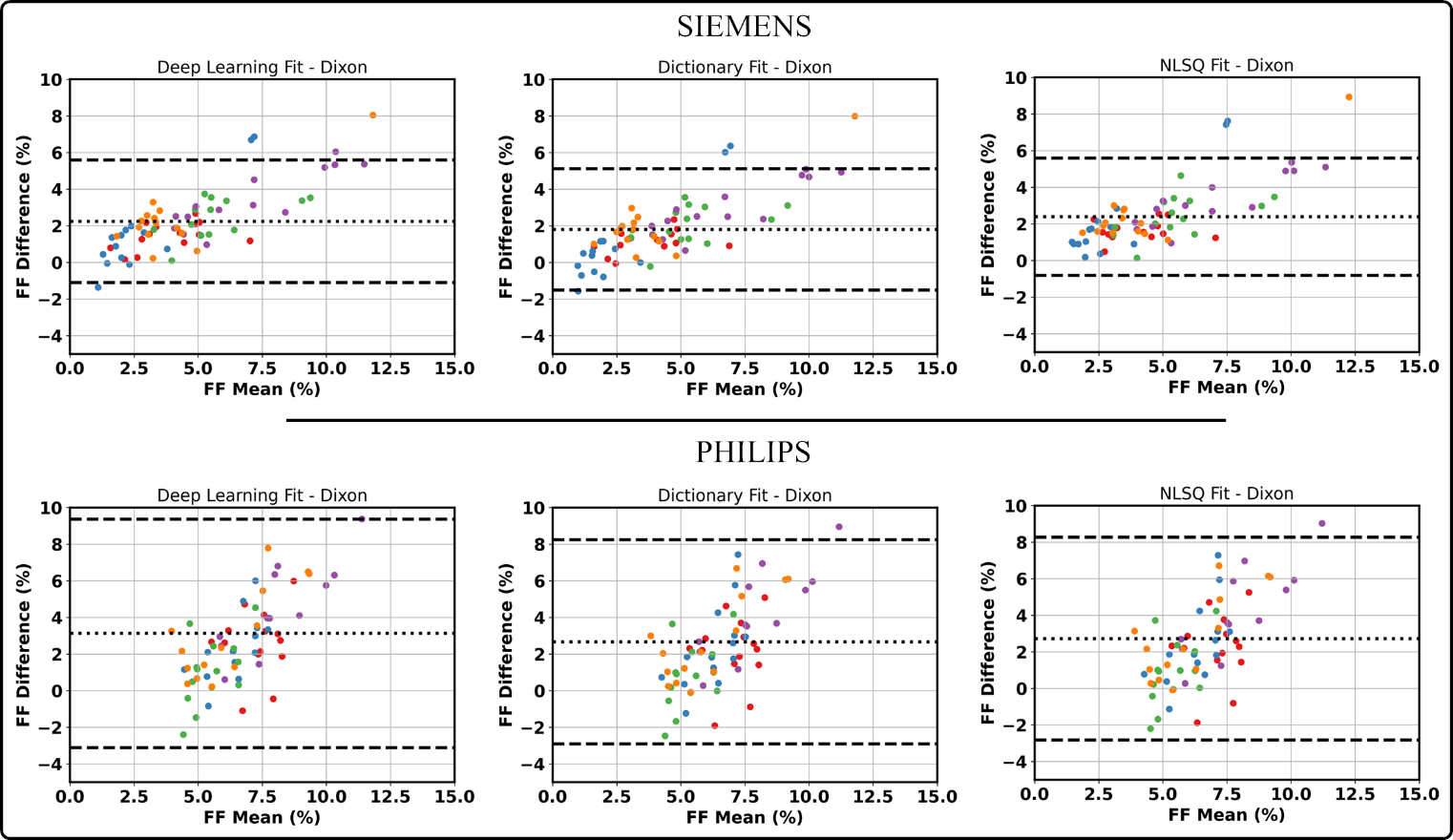


**Figure S3** Bland-Altman plots reporting the comparison of FF values obtained using the EPG approaches with the reference Dixon FF values for data acquired using Simens (top panel) and Philips (bottom panel) scanners.


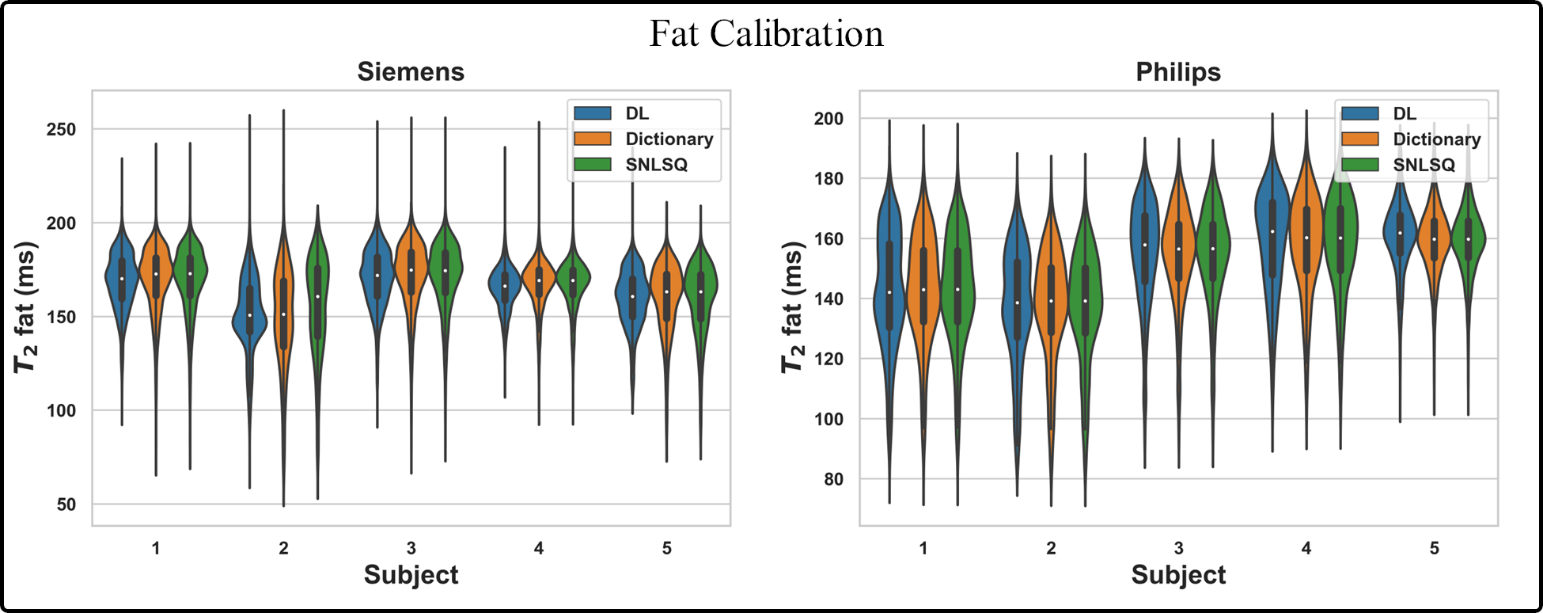


**Figure S4** Violin plots of fat calibration run using different EPG fitting methods for each subject in the Siemens (left) and Philips datasets.


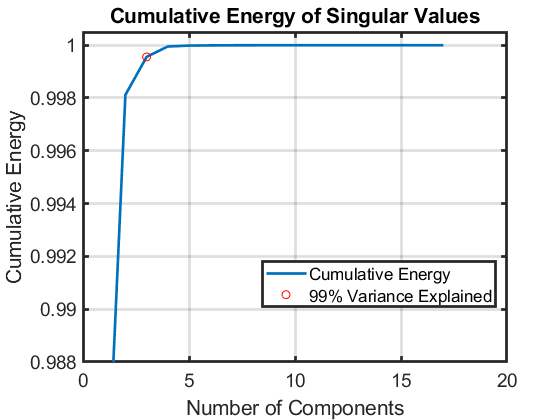


**Figure S5** Cumulative energy as a function of the number of principal components retained along the time-domain of the EPG-simulated MESE dictionary. Three principal components allow to retain more than 99% of the total variance present within the dictionary.
